# Dr. Carl Beck

**Published:** 1911-07

**Authors:** 


### OBITUARY

DR. CARL BECK, of New York, died at his country home at Pelham Heights, Westchester county, N. Y., June 8, 1911, of pneumonia. He was born at Neckargernuend, Germany, April 4, 1856, and received his medical degree at Jena in 1879. He came to New York in 1882, and soon gained prominence as a surgeon, also as an authority in *x*-ray work.

He was professor of surgery and attending surgeon at the New York Post-Graduate Medical School and Hospital and afterward consulting surgeon to the German Polyklinik of New York.

Dr. Beck was inventor of numerous surgical devices and the author of several works in English and German, including Röntgen Ray Diagnosis and Therapy, Surgical Diseases of the Chest and a Textbook on Fractures. His son, Dr. Carl Eric Beck is attending surgeon to St. Mark's Hospital, New York.

